# Relationship between movement behaviours and life satisfaction in Chinese children: A cross-lagged panel analysis

**DOI:** 10.1371/journal.pone.0318735

**Published:** 2025-02-06

**Authors:** Xingyi Yang, Danqing Zhang, Yang Liu

**Affiliations:** 1 School of Physical Education, Shanghai University of Sport, Shanghai, China; 2 Department of Sport, Physical Education and Health, Hong Kong Baptist University, Hong Kong, China; 3 Shanghai Research Centre for Physical Fitness and Health of Children and Adolescents, Shanghai University of Sport, Shanghai, China; Universidad Pablo de Olavide, SPAIN

## Abstract

Understanding the impacts of daily movement behaviours on the well-being of children is crucial for developing effective health promotion strategies. This study examined the relationship between movement behaviours and life satisfaction (LS) using longitudinal data from a sample of primary school students. This one-year follow-up study included 683 students (8.91 ± 1.31 years old) from Shanghai, China. Information on days of moderate- to vigorous-physical activity (MVPA), days of muscle-strengthening exercise (MSE), screen time (ST), and sleep duration was measured via a self-reported questionnaire. Cross-lagged models were used to assess the relationships between movement behaviours at baseline and LS at follow-up. Path analysis showed baseline sleep duration was a significant predictor of subsequent LS. Sex-specific models indicated baseline LS and sleep duration were predictors in males, and baseline MVPA, MSE, and sleep duration in females. Grade-specific models revealed positive relationships of baseline MSE and sleep duration with LS in Level 1 (grade 1 and 2), and baseline MVPA and MSE in Level 2 (grade 3 and 4). This study shows a complex interplay between 24-hour movement behaviours and LS among children. While baseline sleep duration emerged as a consistent predictor of LS at follow-up across the overall sample, the influence of MVPA, MSE, and ST varied by sex and grade level. These results highlight the importance of considering a range of lifestyle factors, including sleep and physical activity, in understanding and potentially enhancing life satisfaction in childhood.

## Introduction

Life satisfaction (LS) is an individual’s cognitive judgment and evaluation of their overall life experience [1]. It is a key component of subjective well-being, alongside positive and negative affect [2]. In recent years, LS has become a central focus in positive psychology research, recognized for its stability beyond transient emotional states, its resilience against circumstantial biases, and its capacity to influence behavioural changes [3–5]. In children, LS is a complex construct, shaped by a range of factors including socioeconomic status, family dynamics, and personal health behaviours [6]. Numerous studies have shown that children with elevated LS tend to do better in many areas. For example, in terms of academic performance, one study showed that the correlation coefficient between children’s academic performance and LS over the same period was around 0.20, and that LS predicted academic performance 5 months later [7]; Correspondingly, Tepordei et al. (2023) reported a positive correlation between academic achievement and LS [8]. Additionally, Guzmán et al. (2019) found that LS mediates the relationship between mental health risks and social functioning, suggesting its pivotal role in enhancing the cognitive development of young individuals [9]. Furthermore, State and Kern (2017) established a link between LS and mental health, with overall LS inversely related to anxiety and depression [10]. This research also indicates a concurrent decrease in behavioural problems. Notably, previous studies have examined the role of physical movement behaviours, such as MVPA and sleep, in determining children’s overall well-being and LS [11–13]. However, these studies often examine these behaviours separately without considering their combined effects.

The World Health Organization (WHO), the Centers for Disease Control and Prevention (CDC), and the Canadian Society for Exercise Physiology (CSEP) all emphasize the importance of healthy movement behaviours, sufficient sleep, and reduced sedentary behaviours for children’s well-being. WHO recommends at least 60 minutes of moderate- to vigorous-physical activity (MVPA) daily and muscle-strengthening exercises (MSE) three times per week for children aged 5–17 [14]. The CDC provides similar recommendations for children aged 6–17, along with age-specific sleep guidelines: 9–12 hours per night for ages 6–12 and 8–10 hours for ages 13–17 [15]. Neither WHO nor CDC offers specific screen time limits. The Canadian 24-Hour Movement Guidelines integrate physical activity, sedentary behaviour, and sleep for children aged 5–17, recommending 60 minutes of MVPA daily, several hours of light activity, 9–11 hours of sleep for ages 5–13, and 8–10 hours for ages 14–17, while limiting recreational screen time to 2 hours per day [16]. This comprehensive framework highlights the combined impact of movement behaviours on health and well-being. Within this context, the 24-hour movement behaviours framework, which includes physical activity (PA), sedentary behaviour, and sleep patterns, is critical. Each element distinctly impacts LS. PA, especially MVPA and muscle-strengthening exercise (MSE), is crucial for enhancing LS, providing physiological benefits and fostering social interactions that contribute to children’s happiness and sense of achievement [17, 18]. These activities improve self-esteem, social skills, and emotional regulation, highlighting the importance of PA in promoting overall well-being. In contrast, sedentary behaviours, particularly excessive screen time (ST), have been associated with lower LS. Prolonged engagement with screens can displace active and social activities, potentially leading to reduced physical fitness and social isolation [19, 20]. This decrease in active engagement can induce feelings of loneliness and contribute to a sedentary lifestyle, negatively impacting LS. The nature and content of ST, often limiting active and social interaction, further affect children’s physical health and social skills. Sleep is another critical factor influencing children’s emotional and cognitive well-being, and consequently, their LS [21]. Adequate sleep is vital for mood regulation and cognitive performance. Insufficient or poor-quality sleep can lead to emotional instability and concentration issues, adversely affecting LS [22, 23]. Blackwell et al. (2020) emphasizes that better sleep quality in children is associated with lower levels of stress and better general health. These factors, in turn, are associated with higher levels of LS [21].

While previous studies have highlighted the importance of these movement behaviours in fostering LS in children, they have often focused on these behaviours in isolation, neglecting their combined effect. Moreover, there is a lack of longitudinal studies that explore these relationships over time, particularly how early levels of these behaviours can predict changes in LS. This gap is crucial, as understanding the dynamic and bidirectional influences between movement behaviours and LS can provide deeper insights into the developmental processes of children. To address these research gaps, the current study will employ cross-lagged panel models (CLPM) to analyse the collected data on 24-hour movement behaviour and LS, aiming to elucidate the directional influences and temporal relationships between these variables over a year. The adoption of CLPM, a prevalent multivariate analysis technique, facilitated the examination of potential relationships between survey variables across different time points. This approach, distinct from other models, allows for the assessment of spurious relationships and clarifies the genuine influences among the variables [24, 25]. This model’s strength lies in its capacity to provide insights into causal relationships over time, offering a significant advantage over traditional static models [24, 25].

The current study aims to explore the intricate relationship between 24-hour movement behaviours and LS among primary school children over a one-year period. It explores how PA, ST, and sleep duration collectively influence LS, considering variations across sex and grade levels. The hypotheses of this study posit that higher levels of PA and adequate sleep positively correlate with LS, while excessive ST negatively impacts it. The novelty of this study lies in its use of CLPM to simultaneously assess how PA, ST, and sleep duration impact LS and how LS, in turn, may influence these behaviours. By elucidating these relationships, the study aims to inform strategies for promoting healthier lifestyles and enhancing LS in children, contributing significantly to the fields of child development and behavioural health.

## Materials and methods

### Study participants

This longitudinal study targeted students enrolled in grades 1 through 4 in Shanghai’s primary schools. Given the primary education system in Shanghai spans from grade 1 to 5 and the necessity for a year-long follow-up, the study was confined to students in grades 1 to 4. Shanghai had a total of 680 primary schools in 2021. For this study, five schools were contacted, and three schools agreed to participate. These three schools were located in two different administrative districts of Shanghai and were selected using convenience sampling based on their willingness to participate. These schools were contacted directly, and their participation was confirmed after discussions with school administrators.

The target sample size was based on the complexity of the cross-lagged panel model, requiring 10 to 20 participants per free parameter. With approximately 25 free parameters, the required sample size was estimated at 250–500 participants. To account for attrition, this was increased by 20%, resulting in a recommended sample size of 600 participants. Within each school, we initially targeted 4 (grades) × 3 (classes per grade) × 40 (students per class), for an estimated total of 1440 potential participants. After the follow-up and data cleaning processes, a final sample of 680 students was retained for analysis, which exceeds the minimum sample size required to achieve sufficient statistical power.

Ethical approval for this study was obtained from the Shanghai University of Sport Scientific Research Ethics Committee. The initial approval (approval number 102772021RT084) covered the baseline data collection, while an additional approval (approval number 102772022RT032) was granted to account for the follow-up component of the study. Consent was fully informed and obtained in written form from both the students and their guardians. Additionally, the principals of the participating schools provided written informed consent on behalf of their institutions. All procedures were conducted in accordance with ethical standards, ensuring the protection of all participants’ rights and privacy.

### Measurements

The survey instrument comprised two sections, encompassing a total of 19 items. The initial section solicited demographic information, including school affiliation, grade, class, student ID, sex, date of birth, parental education levels, and socioeconomic status, amounting to 9 items. The subsequent section probed into the primary variables of interest: a) 24-hour movement behaviours, including MVPA (2 items), MSE (1 item), ST (2 items), and sleep duration (4 items); b) an assessment of LS (1 item).

The MVPA, MSE and ST items were adapted from the "Children and Adolescents Physical Fitness Index Survey Questionnaire (Student Version) [26]." The original questionnaire was developed in Chinese, and as the sample population in this study consisted of Chinese children, no translation was necessary. To reduce the burden on participants, particularly children, only 1 or 2 items from each construct were selected. These items were carefully chosen to reflect the core dimensions of physical activity and sedentary behaviour while ensuring clarity and manageability for younger respondents. The adapted items were validated using the same sample in this study, ensuring that the reliability (CR = 0.943) and structural validity (AVE = 0.615) remained satisfactory for our specific research context [26].

MVPA was assessed by asking, “In the past week, how many days from Monday to Friday did you engage in physical activity for at least 60 minutes?” and “In the past week, how many days from Saturday to Sunday did you engage in physical activity for at least 60 minutes?” PA was defined as “any physical activity that increases your breathing and heart rate,” aligning with the concept of MVPA. Participants responded with a range from 1 = 0 day to 6 = 5 days and from 1 = 0 day to 3 = 2 days, respectively.

MSE was surveyed through the question, "On how many days in the past week did you engage in MSEs such as push-ups, sit-ups, pull-ups, weightlifting, etc.?" Participants responded with a range from 1 = 0 day to 8 = 7 days.

ST was estimated based on "In the past week, how long per day from Monday to Friday did you engage in the following things in your free time? (1) Watching television; (2) Using cell phones, tablets, and other electronic mobile smart terminal products; (3) Using a laptop or desktop computer for Internet chatting, browsing pages, sending and receiving emails, doing homework, etc;" and "In the past week, how long per day from Saturday to Sunday did you engage in the following things in your free time? (1) Watching television; (2) Using cell phones, tablets, and other electronic mobile smart terminal products; (3) Using a laptop or desktop computer for Internet chatting, browsing pages, sending and receiving emails, doing homework, etc;" Participants responded with "1 = none, 2 = 0.5 hours, 3 = 1 hour, 4 = 2 hours, and 5 = more than 3 hours."

Sleep duration was calculated using the first four questions of the Pittsburgh Sleep Quality Index (PSQI), which assess usual bedtime, wake-up time, sleep latency, and sleep duration. Participants were asked to report their average bedtime and wake-up time over the past week, as well as the time it usually takes them to fall asleep. These responses were used to calculate their total sleep duration. The reliability and validity of the PSQI have been extensively validated in the Chinese population, demonstrating its suitability for this study [27].

LS was measured employing the Cantril Ladder, asks respondents to think of a ladder, with the most satisfied life for them being a 10 and the most unsatisfied life being a 0. The single-item life satisfaction scale has shown moderate to high reliability and validity across various samples and cultural contexts [28–30].

Parental education levels and socioeconomic status (SES) were included as covariates in the analysis, alongside sex and age, due to their potential influence on children’s life satisfaction and movement behaviours [31–35]. Parental education levels were assessed using a self-administered questionnaire, where participants reported the highest level of education attained by their father and mother, selecting from the following categories: “Below primary school level”, “Primary school”, “Junior high school”, “High school”, “Post-secondary school”, and “Bachelor level or higher”. Socioeconomic status was evaluated based on participants’ self-perceived economic condition. Respondents were asked to select the category that best described their family’s socioeconomic status from the following options: “Extremely wealthy”, “Relatively wealthy”, “Average”, “Not very well off”, and “Not at all well off”.

### Data collection

To mitigate common method biases, procedural controls were implemented throughout the survey process. Participants were assured of the survey’s anonymity and confidentiality prior to distribution. During administration, students were encouraged to respond independently, with surveyors and teachers providing clarification on queries without influencing responses.

The survey was administered using the “https://www.wjx.cn/” platform. In the classroom, students were provided with a personal ID and the questionnaire link, which they then completed at home with parental assistance. The survey could be accessed and completed using any internet-enabled device, such as a computer, tablet, or smartphone. The initial survey (baseline) was conducted between 08/06/2021 and 14/06/2021, and the follow-up survey was administered from 15/06/2022 to 21/06/2022. For the follow-up, school teachers facilitated contact with students or their parents to ensure participation. Out of 879 collected questionnaires, 683 were deemed valid, yielding a validity rate of 77.70%.

### Statistical analysis

Data entry and matching of two tests were performed using Excel 2016, followed by importing the data into SPSS 24.0 for descriptive analysis. Numerical variables were described using means ± standard deviation, while frequencies (percentages) were utilized for categorical variables. Missing data were handled by listwise deletion to ensure the integrity of the analyses. Subsequently, the Amos software was employed to model the cross-lagged relationships among MVPA, MSE, ST, sleep duration, and LS.

In the cross-lagged panel model (CLPM), each variable (MVPA, MSE, ST, sleep duration, and LS) was treated as both a predictor and an outcome at different time points. The autoregressive paths were specified to capture the stability of each variable across two time points (e.g., MVPA at Time 1 predicting MVPA at Time 2), while the cross-lagged paths were included to examine the reciprocal effects between different variables over time (e.g., MVPA at Time 1 predicting LS at Time 2, and vice versa).

In addition to constructing models for the overall sample, models for samples of different sex and grade levels were considered, with covariates such as sex, age, parental education, and socioeconomic status included to control for their potential confounding effects. During model construction, the Bollen-Stine bootstrap method was applied to address biases in multivariate normality, enhancing the validity of the model assessment, particularly in smaller subsamples [36]. Additionally, 5,000 resamples were generated via bootstrap methods, enabling precise determination of 95% confidence intervals for indirect effects [37, 38]. The structural equation model’s integrity is further reinforced by established metrics including the model goodness-of-fit index (GFI), adjusted goodness-of-fit index (AGFI), comparative fit index (CFI), and root-mean-square error of approximation (RMSEA), comprising a robust validation framework. The structural equation model’s fit was assessed using GFI and AGFI (≥ 0.90 for good fit), CFI (≥ 0.95 for excellent, ≥ 0.90 for acceptable), and RMSEA (≤ 0.06 for excellent, ≤ 0.08 for acceptable). Statistical significance was set as *p* < 0.05 (two sided).

In our analysis, the structure of the model, with its complex relationships and multiple paths, led to fit indices such as Chi-square (χ^2^), TLI, and SRMR indicating a perfect fit due to minimal degrees of freedom. Given the complexity and specific characteristics of our data, these indices are less informative in this context. Therefore, we focused on alternative fit indices (GFI, AGFI, CFI, RMSEA), which are more appropriate for assessing model performance under these conditions and demonstrate a satisfactory fit to the data.

## Results

Table 1 presents the study participants’ sociodemographic characteristics, including 683 children with an average age of 8.91 ± 1.31 years. The sample was nearly equally distributed by sex, with males constituting 50.95% and females 49.05%. By grade level, 51.24% were in Level 1 (grades 1 and 2), while 48.76% were in Level 2 (grades 3 and 4). Parental education levels varied, with the majority of mothers (49.19%) and fathers (45.83%) holding a post-secondary education. In terms of socioeconomic status, the majority of the sample (57.83%) was categorized as average, followed by those not very well off (25.62%).

**Table 1:** Sociodemographic characteristics of the sample (n = 683).

	Mean/Frequency	SD/%
**Age (years)**	8.91	1.31
**Sex**		
Male	348	50.95
Female	335	49.05
**Grade Level**		
Level 1	350	51.24
Level 2	333	48.76
**Father Education Level**		
Below primary school level	4	0.59
Primary school	12	1.76
Junior high school	30	4.39
High school	120	17.57
Post-secondary school	313	45.83
Bachelor level or higher	204	29.87
**Mother Education Level**		
Below primary school level	4	0.59
Primary school	13	1.90
Junior high school	40	5.86
High school	113	16.54
Post-secondary school	336	49.19
Bachelor level or higher	177	25.92
**Socioeconomic Status**		
Extremely wealthy	9	1.32
Relatively wealthy	22	3.22
Average	395	57.83
Not very well off	175	25.62
Not at all well off	82	12.01

Table 2 presents the sex and grade level-specific characteristics of 24-hour movement behaviour and LS. Baseline data showed that students, on average, engaged in MVPA for 4.78 days per week and MSE for 3.00 days per week in overall sample. Average daily ST was 90.97 minutes, while the average sleep duration was 539.45 minutes per day. LS yielded a mean score of 8.46 on the scale used. At the one-year follow-up, the overall students engaged in MVPA for 4.70 days per week and in MSE for 2.46 days per week, on average. Follow-up data indicated an increase in average daily ST to 176.89 minutes and an extension of sleep duration to 560.75 minutes per day. LS score at the follow-up was 8.41.

**Table 2:** Characteristics of 24-hour movement behaviour and life satisfaction.

	Overall (n = 683)		Male (n = 348)		Female (n = 335)		Level 1 (n = 350)		Level 2 (n = 333)	
	Mean	SD	Mean	SD	Mean	SD	Mean	SD	Mean	SD
MVPA at Baseline (days/week)	4.78	1.96	4.74	2.01	4.83	1.91	5.11	1.92	4.44	1.94
MSE at Baseline (days/week)	3.00	2.43	3.00	2.47	3.00	2.40	3.69	2.53	2.28	2.11
Screen Time at Baseline (min/day)	90.97	75.70	97.17	84.90	84.54	64.28	97.57	88.14	84.04	59.27
Sleep Duration at Baseline (min/day)	539.45	48.71	537.72	51.48	541.25	45.65	547.53	54.95	530.96	39.47
Life Satisfaction at Baseline	8.46	1.87	8.31	2.03	8.61	1.68	8.53	2.11	8.38	1.59
MVPA at Follow-up (days/week)	4.70	2.02	4.69	2.03	4.71	2.01	4.67	2.12	4.74	1.90
MSE at Follow-up (days/week)	2.46	2.18	2.50	2.20	2.42	2.15	2.49	2.24	2.43	2.11
Screen Time at Follow-up (min/day)	176.89	117.29	175.20	111.63	178.66	123.03	177.91	117.57	175.83	117.16
Sleep Duration at Follow-up (min/day)	560.75	39.10	559.28	38.26	562.27	39.95	569.59	34.99	551.46	41.04
Life Satisfaction at Follow-up	8.41	1.51	8.39	1.46	8.44	1.57	8.48	1.38	8.34	1.63

Note: MVPA = Moderate- to Vigorous-intensity Physical Activity; MSE = Muscle-Strengthening Exercise

**Fig 1:**
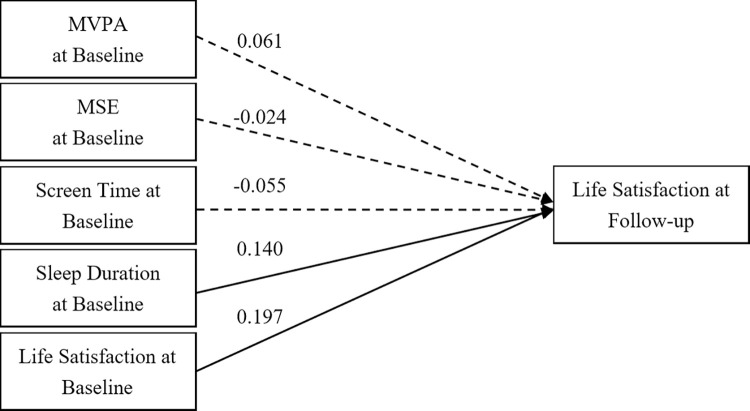
A cross-lagged model of 24-hour movement behaviour and life satisfaction in the overall sample. Note: MVPA = Moderate- to Vigorous-intensity Physica Activity; MSE = Muscle-Strengthening Exercise; Solid lines show significant path coefficients, dashed lines show non-significant path coefficients.

Path analysis of the overall sample (Fig 1) indicates that baseline LS and sleep duration significantly predicted LS at follow-up, with coefficients of 0.197 (*p* < 0.001) and 0.140 (*p* < 0.001), respectively. However, MVPA, MSE, and ST did not show significant predictive paths. The fit indices of the model were excellent, with a GFI of 0.98, AGFI of 1.00, CFI of 1.00, and RMSEA of 0.02.

**Fig 2:**
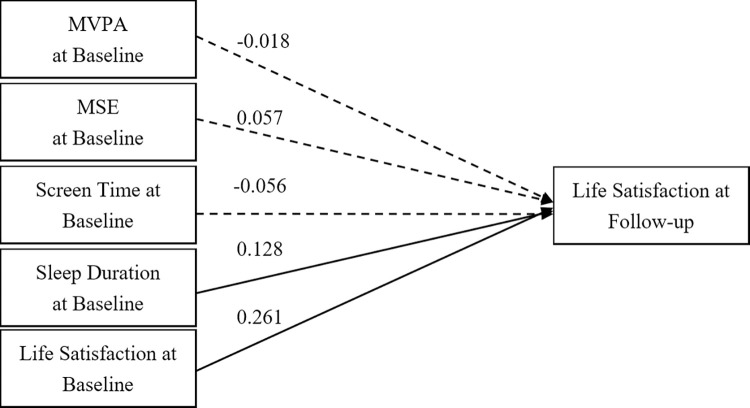
A cross-lagged model of 24-hour movement behaviour and life satisfaction in male sample. Note: MVPA = Moderate- to Vigorous-intensity Physical Activity; MSE = Muscle-Strengthening Exercise; Solid lines show significant path coefficients, dashed lines show non-significant path coefficients.

**Fig 3:**
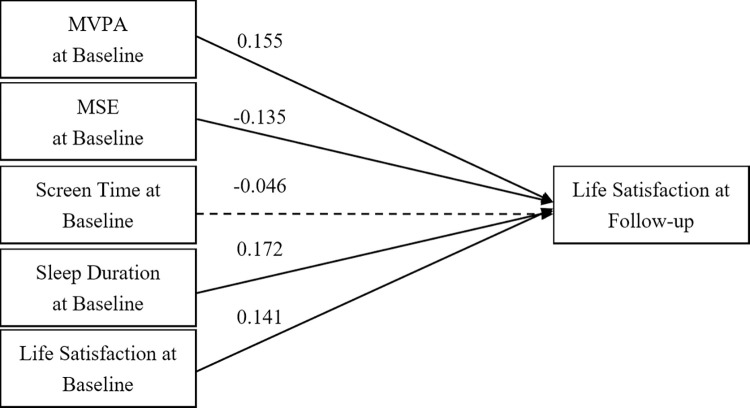
A cross-lagged model of 24-hour movement behaviour and life satisfaction in female sample. Note: MVPA = Moderate- to Vigorous-intensity Physical Activity; MSE = Muscle-Strengthening Exercise; Solid lines show significant path coefficients, dashed lines show non-significant path coefficients.

Figs 2 and 3 present cross-lagged models for males and females, respectively, illustrating predictive relationships between baseline movement behaviours and subsequent LS. In males (Fig 2), baseline LS and sleep duration positively influenced subsequent LS, with coefficients of 0.261 (*p* < 0.001) and 0.128 (*p* = 0.016). However, MVPA, MSE, and ST did not show significant predictive paths. For females (Fig 3), baseline LS, MVPA, MSE, and sleep duration significant predicted later LS, with coefficients of 0.141 (*p* = 0.010), 0.155 (*p* = 0.005), -0.135 (*p* = 0.018), and 0.172 (*p* = 0.001), respectively. Only ST did not show significant predictive paths among females. Both models displayed good fit indices. For males, the GFI was 0.97, AGFI was 1.01, CFI was 0.99, and RMSEA was 0.03. For females, the GFI was 0.97, AGFI was 1.01, CFI was 1.00, and RMSEA was 0.03.

**Fig 4:**
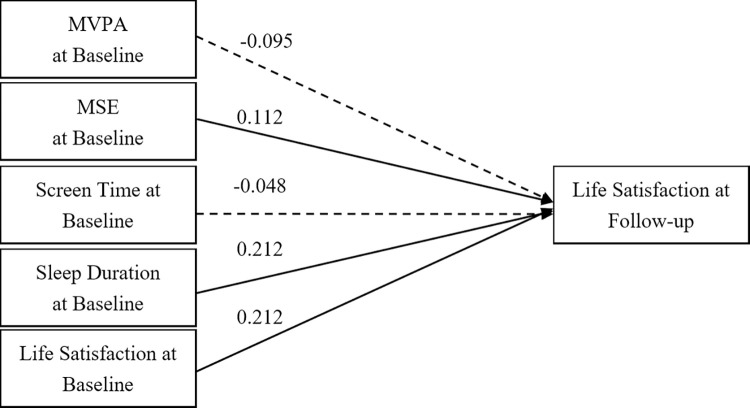
A cross-lagged model of 24-hour movement behaviour and life satisfaction in level 1 sample. Note: MVPA = Moderate- to Vigorous-intensity Physical Activity; MSE = Muscle-Strengthening Exercise; Solid lines show significant path coefficients, dashed lines show non-significant path coefficients.

**Fig 5:**
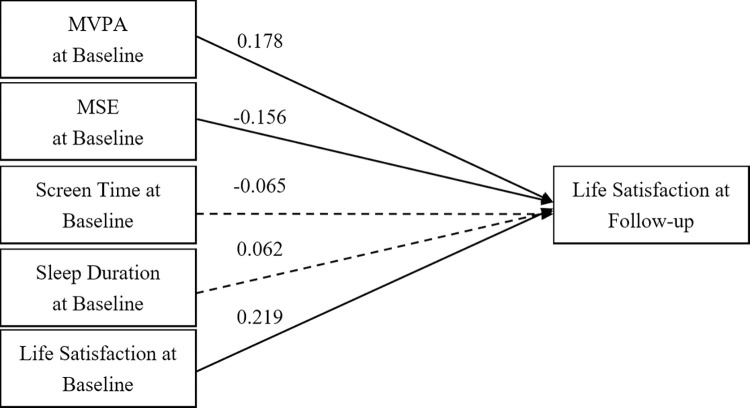
A cross-lagged model of 24-hour movement behaviour and life satisfaction in level 2 sample. Note: MVPA = Moderate- to Vigorous-intensity Physical Activity; MSE = Muscle-Strengthening Exercise; Solid lines show significant path coefficients, dashed lines show non-significant path coefficients.

Figs 4 and 5 illustrate cross-lagged models for Level 1 and Level 2 grades, respectively, demonstrating the predictive influence of baseline behaviours on subsequent LS. The Level 1 model (Fig 4) showed significant positive correlations between baseline LS, MSE, and sleep duration with follow-up LS, with coefficients of 0.212 (*p* < 0.001), 0.112 (*p* = 0.038), and 0.212 (*p* < 0.001). However, MVPA and ST did not show significant predictive paths. The Level 2 model (Fig 5) presented coefficients of 0.219 (p < 0.001) for baseline LS, 0.178 (*p* < 0.001) for MVPA, and -0.156 (*p* = 0.005) for MSE. However, ST and sleep duration did not show significant predictive paths. Both models displayed robust fit indices, with the Level 1 model reporting a GFI of 0.97, AGFI of 1.01, CFI of 0.99, and RMSEA of 0.03, and the Level 2 model featuring a GFI of 0.97, AGFI of 1.01, CFI of 1.00, and RMSEA of 0.02.

## Discussion

This longitudinal study employed a cross-lagged panel analysis to investigate the reciprocal relationships between movement behaviours (MVPA, MSE, ST, and sleep duration) and LS in children over time. The analysis revealed that baseline LS and sleep duration were significant predictors of LS at a one-year follow-up for the overall sample. The cross-lagged model further highlighted sex-specific differences, showing that in males, future LS was predominantly influenced by baseline LS and sleep duration, while in females, a broader range of factors—including baseline LS, MVPA, MSE, and sleep duration—played a significant role. Additionally, the model demonstrated age-specific differences, indicating that in younger children (Level 1), LS, MSE, and sleep duration were positively correlated with future LS, whereas in older children (Level 2), baseline LS and MVPA emerged as more influential factors.

### MVPA

The cross-lagged analysis showed no significant predictive relationship between MVPA and LS in the overall sample, suggesting that other moderating factors might influence the relationship. This finding is consistent with Bae et al. (2017) who did not find a significant relationship between vigorous PA and LS [39]. This finding could be due to a variety of factors, including individual differences in interests, physical abilities, and personal preferences for types of activities. According to Headey et al. (2014), children’s happiness and LS are more closely linked to familial and social relationships rather than specific exercise types [40]. While numerous studies indicated that PA generally correlates positively with LS [11, 13], they often examine this relationship in isolation, neglecting the potential impact of ST and sleep duration on LS. The beneficial influence of MVPA on LS could be counterbalanced by the detrimental effects of excessive ST and inadequate sleep duration. This may also be attributed to differences in measurement methods. For instance, although both Alsubaie (2021) and this study utilized self-reported questionnaires to measure PA and LS, Alsubaie categorized responses to PA into three groups (physically inactive, moderately active, and highly active) and bifurcated LS responses into two categories ("satisfied" and "unsatisfied") [11]. In contrast, this study employed an eight-level self-reported scale (PA: 0–7 days) and an eleven-level scale (LS: 0–10 points). Therefore, variations in the design and granularity of the scales likely contributed to differing findings regarding the relationship between PA and LS.

When looking at sex- and grade- split, the relationship between MVPA and LS becomes more distinct. For females, MVPA positively influenced LS, whereas the influence was not significant in males. Similarly, in the study of Wold et al. (2013) [41], females benefited more than males in soccer. This could be attributed to the social and emotional benefits of PA, such as improved self-esteem, body image, perceived health and social interaction, which are particularly significant for females [41–43]. Furthermore, males and females may engage in physical activities with different motivations and levels of engagement, where males might prioritize competition and physical prowess, and females might seek social interaction and emotional well-being [44]. In older children (Level 2), a similar positive trend was observed. However, this positive association was not as pronounced in younger children. Older children might derive more satisfaction from MVPA due to increased autonomy, the development of specific skills, and the social aspects of participating in sports and physical activities [45, 46].

### MSE

The cross-lagged model indicated that MSE did not significantly predict LS, suggesting a limited direct impact of these activities on children’s life satisfaction. Currently, less evidence can be used to prove the relationship between MSE and LS in children. The lack of a significant predictive relationship between MSE and LS among children can be attributed to several factors. Firstly, children’s perception of LS is influenced more by social and environmental factors than by specific types of PA. Furthermore, the enjoyment factor in physical activities is a significant determinant of LS among children. Activities that are perceived as fun and engaging are more likely to contribute to their overall happiness, as opposed to structured MSE, which might not always be enjoyable for them [47]. Lastly, according to the recommendations of the 24-hour Movement Guidelines, children and adolescents aged 5–17 should engage in MSE for no less than three days per week [16]. However, in the follow-up tests, the average MSE frequency for all children was 2.46 days. This may have resulted in an insufficient positive impact of MSE, thereby leading to a non-significant path coefficient between MSE and LS.

When dissected by sex, the relationship between MSE and LS showed notable differences. Specifically, for females, MSE was negatively associated with LS. This could be due to several factors. Firstly, social expectations and stereotypes around physical activities for females might influence their enjoyment and engagement in MSE [48]. Secondly, the competitive nature of these exercises could impose stress and pressure, particularly on females, affecting their overall LS [49]. This suggests that the way MSE is structured and perceived by females could be crucial in determining its impact on their well-being. The study also revealed that the impact of MSE on LS varied significantly between younger (Level 1) and older (Level 2) children. In younger children, MSE positively correlated with LS. This age group might perceive MSE more as play and an opportunity for physical exploration, which can be enjoyable and contribute to a sense of achievement [50]. In contrast, for older children, the relationship between MSE and LS was negative. As children grow, MSE might become more structured, competitive, and performance-focused, potentially leading to increased pressure and reduced intrinsic enjoyment, thereby negatively impacting LS. Further study should clarify the great difference in the relationship between MSE and LS.

### ST

The cross-lagged analysis found no significant relationship between ST and LS across sexes and grade levels, indicating that screen time may not critically affect life satisfaction. The impact of ST on LS likely varies widely based on the type of screen activity (educational, entertainment, social interaction), the context (alone, with family or friends), and individual differences in preferences and tendencies towards screen use [51–53]. Furthermore, ST only exerts a negative impact on mental health when exceeding 120 minutes [53]. However, in the baseline assessment of this study, the average ST among students was 90.97 minutes, significantly below this threshold, potentially resulting in no substantial effect on LS.

### Sleep duration

The cross-lagged model identified a positive correlation between sleep duration and LS, consistent across all sex and grade subgroups, highlighting sleep’s importance for well-being. This is consistent with previous research, where longer sleep duration is associated with higher LS [54, 55]. This relationship can be attributed to several key factors. Adequate sleep is essential for maintaining good physical health in children, including growth, immune function, and metabolic regulation. The secretion of growth hormones during sleep, particularly in children, contributes to better physical health, leading to increased activity levels and an overall improved quality of life [56, 57]. Furthermore, sleep’s critical contribution to cognitive processes, such as memory consolidation, learning, and attention, leads to improved concentration, problem-solving abilities, and academic performance in children [23]. These cognitive benefits foster a sense of achievement and self-esteem, crucial for LS. Additionally, adequate sleep plays a significant role in mood and emotional regulation, with well-rested children experiencing more stable emotions [21], thus contributing to overall LS. The study’s findings highlight the universal importance of adequate sleep duration, as adequate sleep influences not only emotional regulation and cognitive performance but also social interactions and behaviours. Children with sufficient sleep are better equipped for social engagement, reducing conflicts and enhancing social competence, which are essential for LS [57, 58]. Moreover, chronic sleep deprivation can lead to long-term health issues, including obesity, diabetes, and cardiovascular problems [59], underscoring the need for establishing good sleep habits in childhood to promote healthier lifestyles and reduce the risk of chronic diseases, thereby contributing to higher LS in the long term.

### Study strengths and limitations

The application of cross-lagged panel analysis allowed us to explore the reciprocal and temporal relationships between movement behaviours and LS more comprehensively, offering a nuanced understanding of how these factors interact over time in different subgroups of children. However, the study faces limitations, including its convenience sampling from three Shanghai primary schools, limiting broader applicability. The reliance on self-reported data for PA, ST, and sleep duration could introduce response biases, and the single-item measure for LS, despite its reliability and validity, may not fully capture the multidimensional nature of this construct. Furthermore, the limited number of time points reduces the ability to establish causality, and there is a potential influence of unmeasured variables such as cultural factors, mental health status, and environmental influence.

A key limitation of cross-lagged analysis is its reliance on correlational data, which cannot establish definitive causality and may be influenced by unmeasured confounders. The method assumes linear and stable relationships over time, which may not capture the complexity of real-life dynamics. Additionally, the chosen time intervals may not align perfectly with the actual changes, potentially biasing the estimates. To address these limitations, we selected appropriate time intervals and controlled for key covariates, but future studies could use longer follow-up periods, more measurement points, and broader covariates to better capture these dynamics.

## Conclusion

In conclusion, this longitudinal study reveals the varied associations between 24-hour movement behaviour and LS in children. Specifically, sleep duration emerged as a consistent predictor of LS across all groups, underscoring its crucial role in children’s well-being. While MVPA and MSE did not show a significant relationship with LS in the overall sample, sex-specific differences were observed. This suggests that the influence of physical activities on LS may vary based on sex. Additionally, ST did not show a significant predictive relationship with LS, indicating a more complex interplay than commonly perceived. The study highlights the importance of considering individual differences and developmental stages when assessing the impact of movement behaviours on children’s well-being, providing valuable insights for tailored interventions and policies.
